# The Effects of Alcohol Hangover on Mood and Performance Assessed at Home

**DOI:** 10.3390/jcm9041068

**Published:** 2020-04-09

**Authors:** Chris Alford, Zuzana Martinkova, Brian Tiplady, Rebecca Reece, Joris C. Verster

**Affiliations:** 1 Department of Health and Social Sciences, University of the West of England, Bristol BS16 1QY, UK; 2 Department of Anaesthesia, Critical Care & Pain Medicine, University of Edinburgh, Edinburgh EH8 9YL, UK; 3 Division of Pharmacology, Utrecht Institute for Pharmaceutical Sciences (UIPS), Utrecht University, 3584CG Utrecht, The Netherlands; 4 Centre for Human Psychopharmacology, Swinburne University, Melbourne, VIC 3122, Australia

**Keywords:** alcohol, hangover, mood, performance, assessment at home, mobile testing

## Abstract

The current study evaluated the next day consequences of a social night of drinking compared to a no alcohol night, with standardised mood and portable screen-based performance measures assessed in the morning at participants’ homes, and a breathalyser screen for zero alcohol. A mixed sex group (*n* = 20) took part in the study. Participants reported consuming on average 16.9 units (135 g) alcohol, resulting in a hangover rating of 60 (out of 100) compared to 0.3 following the no alcohol night. Statistical significance comparisons contrasting the hangover with the no alcohol condition revealed an increase in negative mood and irritability during hangover and an (unexpected) increase in risk and thrill seeking. Performance scores showed an overall slowing of responses across measures, but with less impact on errors. The results support the description of hangover as a general state of cognitive impairment, reflected in slower responses and reduced accuracy across a variety of measures of cognitive function. This suggests a general level of impairment due to hangover, as well as increased negative mood. The use of a naturalistic design enabled the impact of more typical levels of alcohol associated with real life social consumption to be assessed, revealing wide ranging neurocognitive impairment with these higher doses. This study has successfully demonstrated the sensitivity of home-based assessment of the impact of alcohol hangover on a range of subjective and objective measures. The observed impairments, which may significantly impair daily activities such as driving a car or job performance, should be further investigated and taken into account by policy makers.

## 1. Introduction

The negative consequences of alcohol consumption on safety and productivity are well known, but the separate impact of alcohol hangover has historically received less attention. This is changing, and alcohol hangover is now being recognised as important in its own right. Around 9% of workers in the USA report having a hangover whilst at work, which can impair performance both through absenteeism and “presenteeism”—attending but unable to work effectively [1]. There are also wider implications for health and safety [2,3,4]. Safety critical daily activities such as driving have shown impairment with hangover [5,6]. The economic costs of hangover in terms of absenteeism and presenteeism are estimated at 4 billion GBP annually [7].

The alcohol hangover refers to the combination of negative mental and physical symptoms which can be experienced after a single episode of alcohol consumption, starting when blood alcohol concentration (BAC) approaches zero [8,9]. Symptoms reflect a general state of malaise described medically as veisalgia. The most common symptoms may be grouped as follows: (1) drowsiness, including fatigue, sleepiness and weakness; and (2) cognitive problems, including reduced alertness and difficulties with memory and concentration. Other symptoms, such as disturbed water balance, contribute less to the overall hangover [10,11].

A simple “culprit” responsible for this range of symptoms has yet to be identified. The toxic metabolite acetaldehyde has largely gone from the system when hangover remains, although research suggests that an inflammatory response and cytokine elevation may account for some symptoms [12,13,14,15]. Changes in neurotransmitters and mitochondrial dysfunction, as well as the congeners added to different types of drink may also have a role [16]. The presence and severity of a hangover have been linked to the level of prior consumption in some studies, with the proportion of hangover resistant participants reducing to closer to zero as consumption levels achieve 0.3–0.4% estimated BAC [17,18]. However, individual differences affecting metabolism, such as genetic variation in alcohol dehydrogenase, as well as personality and health status, reflect a wide range of reported symptoms and overall severity [13,16]. Sex differences in alcohol metabolism are well known and may also reflect variability of the presence and severity of hangover symptoms in men and women, and their impact on cognitive and psychomotor functioning and mood [19,20]. Surveys generally indicate higher social consumption levels by males, although gender differences in consequential hangover severity have not been consistently reported, and may be more apparent in women at intermediate BAC levels (0.1–0.3%) [21,22].

Acute alcohol consumption tends to lead to an increase in errors, with less effect on speed of performance, possibly reflecting a “risky shift” [23,24]. In contrast, slowed responses seems a more general consequence of alcohol hangover and may reflect the distinct neurocognitive effects when contrasted with alcohol intoxication [25,26]. A key problem facing alcohol hangover research is the need for participants to achieve sufficient levels of alcohol consumption, in order to produce a measurable hangover for impairment studies to be effective. Due to ethics constraints, sufficient alcohol dosing required to achieve higher real-life BACs cannot always be administered in experimental settings; or requires stringent assessment and prolonged post dose participant support. For example, supervised overnight stays at the research facility are required to monitor participants’ wellbeing. This makes these studies resource intensive and demanding for participants [27]. In addition, social drinking frequently includes a mixture of alcoholic beverages being consumed in a single drinking occasion which cannot easily be reproduced in a laboratory. This has led to a number of naturalistic studies where typically residual effects next day are assessed, contrasting a regular drinking night as part of normal social life with a no alcohol consumption night [27]. The resulting hangover assessment can then still include the same validated measures as used in RCTs [27,28]. However, the hangover state in naturalistic studies reflects a real-life hangover experience significantly better than studies that involve lab-based alcohol administration [26,27,29].

The following investigation was based on a naturalistic study design with participants assessed within their own homes in order to maximise their safety and comfort after a potentially heavy social drinking session. The aim of using the naturalistic study design was to mimic real-life as closely as possible and as such is characterised by a minimum of lifestyle rules for participants. The investigators did not (actively) interfere with their activities, and behaviours and activities of the participants were neither standardised nor regulated by the study protocol. All assessments were undertaken whilst participants remained in their usual environment (i.e., at home), which was less demanding for participants and the results may more accurately reflect real life. Balanced gender groups were included to see if there were any marked differences. The principal research questions were to determine whether subjective state and objective performance measures differed when assessed at home the morning after a night of social drinking compared to a night without alcohol. Based on previous research, it was hypothesised that both performance and mood are impaired during alcohol hangover. The secondary exploratory research question was to evaluate possible relationships between measures including reported alcohol consumption and subsequent subjective state and performance.

## 2. Methods

### 2.1. Design

The study was a two-period comparison of performance and subjective state between days following an evening of drinking or an evening with no alcohol. One day in each condition was assessed for each subject. The study was unblinded, and the order of conditions was not specified in advance. The study was approved by the University of the West of England Faculty Ethics Committee (Approval number: HLS130235, 1 March 2013), informed consent was obtained from all participants and the study was undertaken in accordance with the British Psychological Society Code of Ethics and Conduct (2009).

### 2.2. Participants

Participants were social drinkers, with no specific criterion of hangover frequency. They were recruited using the snowball technique from acquaintances of people known personally to the principal experimenter, rather than the direct friends of the experimenter. This approach was used to minimise the impact of personal relationships, whilst optimising researcher safety as well as participant comfort for home-based assessment. Exclusion criteria were frequent illicit drug use, or use prior to testing, any health issues, including those adversely affected by alcohol, breast feeding, pregnancy and those who may be pregnant. No financial payment was made for participation.

Most participants were (friends of) students from the University of the West of England, providing an age range of 18–33 years old. The sample consisted of twenty healthy Caucasian participants, ten female (mean ages of 28.8 years old). Thirty-five per cent were smokers (mean 4–5 cigarettes per day), with 65% in full employment and 5% students. They reported having a mean (SD) of 2.8 (1.3) (range 1–6) hangovers per month.

### 2.3. Procedure

Participants were given information sheets describing the study as well as inclusion and exclusion criteria. Those eligible to be enrolled then provided signed informed consent and were advised of their rights to withdraw. Enrolled participants completed a demographic questionnaire and assigned their unique participant code facilitating anonymity, and reminded that participation and all information was confidential. Participants were reminded not to consume any stimulants, e.g., coffee, tea and chocolate, or smoke an hour before assessment, as well as to make a note of the number and type of drinks they consumed the previous evening for their residual alcohol assessment days.

Participants contacted the investigator to arrange a suitable time for an assessment visit and were tested at their homes in the morning (08:00–12:00) with the experimenter accompanied by a research buddy who remained in the car outside during the visit. Assessment day and treatment order selected by participants resulting in 65% undertaking the hangover condition first. An initial positive breath alcohol test resulted in the schedule start being moved back enabling a zero reading to be obtained, or testing rearranged, so that all assessments were completed with zero BAC% following drinking, as well as in the control condition. Subjective assessments were followed by performance tests with the total assessment period completed within a 45 min period. There was an average of 5 weeks between treatments and minimum washout period of a week following the hangover assessment. Participants were given a debrief sheet including contacts for support and advice about excessive drinking after completing both assessments, and being reminded of their rights to withdraw their data.

### 2.4. Assessments

Breath Alcohol Concentration (BAC%) was assessed with a Lion Alcolmeter 400 (Lion Laboratories, Barry, South Glamorgan, UK) by the experimenter who kept their hand over the visual display so that participants were blind to readings.

Subjective Assessments comprised paper questionnaires including general mood assessment dimensions, namely “alert”, “calm” and “content”, assessed with 100 mm Visual Analog Scales (VAS) [30]; an Irritability Questionnaire based on a 4-point Likert scale [31]; risk taking comprising a 100 mm VAS [32]; the Alcohol Hangover Severity Scale (AHSS) with an 11-point scale, 0–10 [28,33]; and a 1-item overall hangover severity score (a single item, rated on a 100 mm VAS, ranging from “not at all” to “worst ever hangover”) [28]. Participants were also required to record their alcohol consumption on their social nights out with the aid of a retrospective diary when assessed in their homes.

Performance Tests were selected from the Penscreen Test Battery (www.MobileCognition.com) presented on ARNOVA 7 G2 android 7 Inch/17.8 cm Screens. The five a priori selected tests (Arrow Flankers, Continuous Performance, Four Choice Reaction Time, Serial Sevens and Stop Signal Task) each included a single measure of response time and a single error measure for each test to enable a speed–accuracy trade-off evaluation to be undertaken, and with a view to limiting type I errors in the subsequent statistical analyses. Each test was explained to the participant and performed by the researcher where necessary, before the participants had an initial practice, as well as being provided with a printed sheet describing each test including the stimulus display and the required responses. Participants were asked to respond as quickly and accurately as possible, with a practice block preceding each test which took around 5 min to complete with derived test variables including response speed (reaction time) and number of errors, and the total assessment period taking up to 45 min.

Arrow Flankers [34,35] measures divided attention and inhibition. Sets of five symbols appear on the screen one set at a time. The central symbol (target) is always an arrow, pointing either to the right or the left. The other four symbols (flankers) are either congruent (arrows pointing in the same direction as the target); incongruent (arrows pointing in the opposite direction to the target); neutral (squares); or suppressors (crosses). The task is to press a left or right button, corresponding to the central arrow, unless the flankers are crosses, in which case no response should be made. Outcome measures are the mean overall reaction time and the total number of errors.

Continuous Performance [36,37] measures sustained attention. The A–X version of the test was used. Letters appear on the screen one at a time. The task is to respond when X appears immediately following A. Outcome measures are the mean reaction time for correct responses and the number of missed targets.

Four Choice Reaction Time [38,39] is a measure of psychomotor performance. An array of four “lights” on the screen corresponds to four buttons below. Each of the lights is highlighted in turn, and the participant responds by tapping the corresponding button as quickly as possible. Outcome measures are the mean reaction time for correct responses and the number of incorrect responses.

Serial Sevens [39,40] assesses arithmetic and working memory. A starter number in the range 800–899 is presented on the screen followed by a series of descending 3-digit numbers. The task is to tap a Yes key if the number is seven less than the previous number shown. In other cases, participants press “No”. Outcome measures are the mean reaction time for correct responses and the number of incorrect responses.

Stop Signal Task [41,42] is a measure of impulsivity and inhibition. A letter stimulus which is either an O or an X is displayed, together with two on-screen buttons. The task is to tap the left button for an X, the right button for an O, as quickly as possible. In one trial in four, a stop-signal is presented after the onset of the letter, consisting of two horizontal red lines superimposed on the letter display. When a stop-signal appears, the user should not respond to the letter stimulus. Outcome measures are the mean reaction time for correct responses and the number of missed stop signal items (i.e., response not withheld).

### 2.5. Statistical Analyses

Statistical analyses were undertaken using IBM SPSS Version 25 (IBM SPSS Statistics 2015, IBM Corp, Armonk, NY, USA). In line with the research questions, statistical analyses were based on contrasting data from the residual alcohol (alcohol hangover) with the no alcohol condition, as well investigating possible relationships among subjective hangover, the amount of alcohol consumed the previous night, and performance and other subjective measures. Separate MANOVAS were first performed to limit type I errors for the subjective and performance data, including independent variables within groups: measure (performance tests or questionnaires) and alcohol (post-alcohol or no alcohol); and between groups factors: assessment order (post-alcohol or no alcohol first) and gender (male or female). They were rerun as a reduced model (questionnaires) with nonsignificant factors removed (assessment order and gender). Where MANOVA indicated an interaction between alcohol and gender (performance tests), ANOVAS were run including alcohol and gender as factors. Overall significant effects for alcohol enabled subsequent paired comparisons to be run for individual variables (each performance test, or each questionnaire) contrasting no alcohol with the post-alcohol condition. Nonparametric paired comparisons were run as confirmatory tests as a control for departures from normality amongst test variable data and identified the same significant paired contrasts as the parametric analyses. Exact *p* values are reported. Tests of association were run as partial correlations with hangover frequency (monthly) as a control for possible tolerance effects [43] as well as smoking as a control, which may impact hangover [44]. Two-tailed significance levels (*p* < 0.05) were used for all analyses.

## 3. Results

Twenty participants successfully completed the experimental protocol undertaking assessments in their own homes after a night of social drinking (alcohol night) and a night without alcohol consumption (no alcohol). All participants had zero BAC confirmed with the breathalyser prior to their assessments.

### 3.1. Alcohol Consumption

All participants reported zero alcohol consumption on their no alcohol nights before testing, and they reported consuming a mean (SD) of 16.9 (4.2) units (range 8–26), calculated using the UK National Health Service (NHS) website tool (available at: www.nhs.uk) to convert recorded drinks into standard UK units (1 unit = 10 mL, 8 g pure alcohol). This level of consumption is similar to those reported elsewhere including 13.5 standard drinks (10 g pure alcohol) reported in a recent Australian study [26] averaging 135 g pure alcohol, whilst for this study 16.9 units of 8 g/10 mL is also equivalent to averaging 135 g pure alcohol based on the UK definition. Most participants mixed their drinks, with two participants having only one type of drink, most 2–3, and two of the 20 participants mixing four different drinks, typical of a naturalistic study.

### 3.2. Subjective Assessments

Multivariate analysis failed to reveal significant effects for treatment order (η^2^*p* = 0.00) or gender (η^2^*p* = 0.08), including interactions; thus, these factors were removed and data reanalysed with the reduced model where alcohol remained significant (F_(1,19)_ = 17.98, *p* < 0.0001, η^2^*p* = 0.49), reflecting an overall difference in subjective ratings between the alcohol and no alcohol conditions and enabling paired comparisons to be undertaken for the individual measures. The subjective assessments and results of the pairwise statistical analyses are summarised in Table 1.

The parametric analyses provided the same significant contrasts as the nonparametric confirmatory analysis. Significant increases (*p* < 0.0001) were seen following alcohol for both the AHSS and the one-item hangover severity VAS, with both showing close to zero symptom ratings after no alcohol but notable increases after alcohol (see Table 1). For example, a mean (SD) one-item hangover severity VAS scores of 0.3 (1.1) (range 0–100) was reported following no alcohol rising to 60.3 (20.0) (range 15–90) the day after alcohol consumption, confirming overall hangover status amongst participants.

The VAS mood scale revealed a significant reduction in measures of alertness (*p* = 0.002) and calmness (*p* = 0.005) in the hangover condition and a trend for a reduction in contentedness (*p* = 0.075). A significant increase was also recorded with the irritability scale (*p* < 0.0001), reflecting an overall reduction in positive mood and increased negative state with alcohol hangover compared to the no hangover condition. Although no overall change in subjective risk taking was recorded with EVAR, a modest increase was seen in risk and thrill seeking (*p* = 0.032), although no change in need for control.

### 3.3. Performance Tests

Multivariate analysis again failed to find overall significant effects for either treatment order (η^2^*p* = 0.00) or gender (η^2^*p* = 0.10), but did find a significant alcohol × gender interaction (F_(1,16)_ = 8.1, *p* = 0.012, η^2^*p* = 0.34), which was explored with ANOVAs run for each variable and including alcohol and gender as factors. Alcohol × gender interaction was found for serial sevens response time (F_(1,18)_ = 7.1, *p* = 0.016, η^2^*p* = 0.28) and errors (F_(1,18)_ = 8.1, *p* = 0.011, η^2^*p* = 0.31) where males had significantly slower responses (1712.0 (699.9) versus 1005.3 (332.9), *p* < 0.0001) and more errors (6.2 (5.9) versus 3.1 (4.6), *p* = 0.011) after alcohol compared to no alcohol, although no significant effects were found for females. There were no other significant interactions or gender differences. An overall effect for alcohol was seen with response times for arrow flankers (F_(1,18)_ = 5.1, *p* = 0.036, η^2^*p* = 0.22), continuous performance (F_(1,18)_ = 8.1, *p* = 0.011, η^2^*p* = 0.31) and choice reaction time (F_(1,18)_ = 11.5, *p* = 0.003, η^2^*p* = 0.39), and for errors with arrow flankers alone (F_(1,18)_ = 10.1, *p* = 0.005, η^2^*p* = 0.36). These differences were further examined with paired comparisons. Results for the cognitive tests and the pairwise comparisons are summarised in Table 2.

The parametric paired contrasts reflected the nonparametric analyses (see Table 2). Responses were significantly slower with arrow flankers (*p* = 0.036), continuous performance (*p* = 0.011) and choice reaction time (*p* = 0.005). Errors were significantly increased in the hangover condition for arrow flankers (*p* = 0.004).

### 3.4. Associations between Measures

The secondary research question was concerned with possible associations between subjective state including perceived hangover, performance and other variables. Statistical analysis employed partial correlations with monthly hangover frequency to take into account possible tolerance effects [43] and smoking habit (categorised as yes, occasional, no) [44], included as control variables which may impact scores in the assessed hangover condition).

The one-item overall hangover severity score was selected as an overall measure of subjective hangover and was found to be more sensitive than the AHSS [28], with a relatively weak association between the two scales (r = 0.439, *p* = 0.068). Overall hangover severity (one-item) correlated significantly with the recalled amount of alcohol consumed the night before (r = 0.548, *p* = 0.019), with the more alcohol consumed the previous evening the greater the subjective hangover the following day. The amount of alcohol consumed the previous evening failed to show any significant associations with performance or other subjective variables. However, overall hangover severity was also correlated with other performance and subjective data collected in the hangover condition.

With the VAS, increased one-item overall hangover severity was associated with participants feeling less content (r = −0.524, *p* = 0.026). Risk taking (EVAR) indicated a reduction in the self-confidence subscale with increased hangover (r = −0.493, *p* = 0.037), but an unexpected increase in the risk and thrill seeking subscale (r = 0.479, *p* = 0.044).

There was a significant association between the 1-item overall hangover severity score and cognitive performance. With serial sevens (continuous subtraction), increased hangover was unexpectedly associated with faster responses (r = −0.623, *p* = 0.006) and fewer errors (r = −0.541, *p* = 0.020), although there were no other significant associations. In contrast to the one-item overall hangover severity score, none of the associations among mood, performance and AHSS scores were significant.

## 4. Discussion

The main aim of the study was to investigate differences in both subjective state and performance following a night of social drinking that may result in a hangover next day when assessed at home and contrasted with a night without alcohol consumption using a naturalistic design. A relatively high mean (SD) alcohol consumption was reported of 17 (4.2) units (range 8–26) based on primed recall next day, and with most participants consuming two or more types of drinks. Mixing drinks, or the order in which they are consumed, has not in itself shown an impact on hangover [45], although congeners may have a role in determining hangover severity [46]. The mean (SD) hangover severity score of 60.3 (20.0) on a one-item scale ranging from “not at all” (0) to “worst ever” (100) reflects a clearly demonstrable hangover for this group overall, although BAC was not directly measured. Significant differences in the present study were associated with overall effect sizes (η^2^*p*) of around of 0.5 for subjective measures and 0.2–0.4 for performance measures. These are comparable with those reported in compatible repeated measures studies [47], and supported by impairments reported in other measures such as driving [5], suggesting the real life consequences of alcohol hangover after social drinking occasions in everyday life.

### 4.1. Subjective Measures

Six of the 10 subjective measures returned significant differences between the alcohol hangover and no hangover conditions (see Table 1) showing that participants were clearly experiencing different subjective states, and the reduction in alertness is consistent with the study of 1400 participants identifying key factors of the subjective hangover state [11]. The reduction in positive mood measured with the VAS and increase in irritability supports the descriptions of hangover as a state of malaise and supported by significant increases in both specific hangover symptoms measured with the AHSS as well as the overall one-item hangover severity score.

The finding of a significant positive association between recalled consumption the previous night and one-item hangover severity assessed next day (r = 0.548, *p* = 019) has been found in some other studies but not all, and thus the relatively small sample size included here provides limited support for this [26]. Given the wide range of hangover symptoms including CNS, gastrointestinal and more general physiological effects [11,48], as well as individual differences in alcohol metabolism that may impact hangover including reported immunity, larger samples are required to establish this association, possibly with selected cohorts where distinct groups of phenotypes are found. The average change in subjective hangover (one-item severity score) ratings from less than 1 on the control day to 60 (out of 100) on the hangover day indicates a notable hangover for the majority of participants. The AHSS also increased significantly, but a relative lack of sensitivity for the AHSS when correlated with other measures may reflect its component nature, where a restricted number of individual symptoms may not reflect the complete hangover experience and its impact accurately [28]. The decrease in positive mood with the VAS supports the hangover ratings, with significant reductions in feeling alert and calm, and a trend for a reduction in feeling content in the hangover condition compared to no alcohol, and supports the reliability of the subjective data.

The EVAR scale has been widely used in assessing risk taking and found to be a sensitive and reliable measure [32]. The component scores showed a differential effect when contrasting hangover with no alcohol conditions. Overall risk taking, need for control and self-confidence were relatively unchanged. Interestingly, risk and thrill seeking were increased which would not be predicted for participants in a hangover state that may be associated with increased feelings of fragility and vulnerability. However, reduced executive function [25] may result in disinhibition during hangover that may then be reflected in increased risk and thrill seeking ratings. This has been seen alongside increased impulsivity with alcohol and driving [49], although the stop signal task failed to show significant changes in the present study, suggesting a different impact profile for neurocognitive domains linked to risk taking and impulsivity behaviour with hangover.

Although recalled alcohol consumption was associated with one-item overall hangover severity, other measures were not, suggesting a lack of direct changes in mood states with quantitative changes in alcohol consumption and resulting hangover status. However, hangover severity did correlate significantly with decreases in self-confidence and contentedness, as well as increased risk and thrill seeking, suggesting the sensitivity of subjective awareness of a hangover state. These results contrast with the more limited associations found between subjective hangover and performance measures.

### 4.2. Performance Tests

The five performance tests assessed a range of cognitive functions including psychomotor performance (choice reaction time), sustained attention (continuous performance) and executive functions. All tests included a response time measure as well as errors enabling a potential speed—accuracy trade-off to be evaluated. Executive function measures included working memory (serial sevens) as well as inhibition (arrow flankers and stop signal task). In contrasting the no alcohol and hangover conditions, four of the five tests indicated a significant contrast (arrow flankers, choice reaction time, continuous performance and (for males only) serial sevens), reflecting impaired performance with slower responses with hangover. Significantly increased errors were only seen with arrow flankers and (for males only) serial sevens, although more errors were generally seen in the hangover condition. The stop signal task alone failed to show differences (see Table 2).

Slower response speeds were evident for all but the stop signal task, resulting in significant slowing with hangover across test averages, although for serial sevens the difference was only significant for male participants. Increased errors only achieved significance with arrow flankers and (for males only) serial sevens, but all except the stop signal task showed an increased number of errors with hangover. These results are in opposition to the speed–accuracy trade-off that has been reported for alcohol, where response speeds are maintained but with an increase in errors [23]. This may be associated with a “risky shift” after acute alcohol administration and observed in the field [50] and characteristic of disinhibition. The speed—accuracy trade-off has not always been observed and error rates can also increase with alcohol induced impairment [51]. However, based on the overall profile of significant results observed in this study, response slowing is a more consistent feature of alcohol hangover induced impairment than increased errors and this has been observed in other studies [26,52,53]. The greater impact on response slowing is therefore an emerging important factor in profiling alcohol hangover induced impairment in contrast to the effects of acute alcohol intoxication.

The overall results of slower response and more errors for all except for the stop signal task, supports alcohol hangover being associated with impairment and slowing of cognitive functions. The apparently contradictory finding of a significant association with serial sevens (assessing mental arithmetic, working memory) and increased hangover severity with faster and more accurate responses, albeit within a general slowing of responses of 100 ms between the no alcohol and hangover conditions, was unexpected. This might reflect an increased situational awareness [54] of being in a sensitive state with increased perception of hangover and possibly increased effort as a result, but was found for this performance measure alone and requires replication.

### 4.3. Limitations and Strengths

Study limitations included the limited number of participants (*n* = 20) and, although this was sufficient to show both differences between the no alcohol and alcohol hangover conditions, which has been supported by a partial replication, as well as some associations between measures, it was underpowered for gender comparisons and a more reliable investigation of associations between measures. The age range of participants was 18–33 years old. Although high level drinking and hangovers are frequently reported by this age range, future studies should examine to what extent the current findings can be generalised to other age groups. Especially, hangover data of elderly are lacking. Whereas declines are observed in overall drinking in the last 15 years [55] and a growing number of British young adults routinely do not drink alcohol [56], reported alcohol use and corresponding problems in older age groups are reported to be on the increase [57,58].

The naturalistic design did enable the impact of higher alcohol consumption levels (17 units) to be assessed reflecting real life. This enabled a number of differences in both subjective and performance measures to be found between the hangover and no alcohol conditions, suggesting an overall successful investigation in relation to the primary research question. The partial replication also supports these general findings. Assessment at home provided a more natural environment to record actual subjective state and functionality, as well as improving participant safety if travelling in a potentially vulnerable condition. In addition, where participants initially showed a small positive BAC with breathalyser the morning after drinking, assessments could be comfortably delayed until a zero BAC was recorded. These attributes of home-based assessment enabled all participants to complete the study protocol without dropping out, suggesting that this approach places less demands on participants and is therefore a further benefit of naturalistic designs in alcohol hangover research [27]. These differences may account for some of the observed differences in alcohol hangover studies when comparing lab with naturalistic studies. However, one study which directly compared the effects of acute alcohol consumption in the laboratory and in a naturalistic setting showed similar patterns of impairment by alcohol in both settings [59].

## 5. Conclusions

This study employed a naturalistic design so that participants were able to reflect a typical hangover state after a night out of social drinking with higher consumption, unrestrained from laboratory protocol and ethical limitations on alcohol administration. The use of home-based assessment was successful, demonstrating significant effects on both mood and performance. The pattern of impairment found here supports other findings of alcohol hangover as inducing a state of malaise or veisalgia which negatively impacts a range of cognitive abilities including executive functions, and in addition to more negative mood and experience of hangover symptoms. The findings support others showing both mood and performance impairment with alcohol hangover, and a pattern of general impairment is emerging that is distinct to that found in acute alcohol administration studies. Future studies should determine how the observed impairments may have negative consequences in everyday life, work performance and daily activities such as driving.

## Figures and Tables

**Table 1:** Subjective assessments of mood and hangover.

Measure	Control	Alcohol
Alert VAS (0–100)	51.2 (4.9)	44.1 (6.8) *^,†^
Content VAS (0–100)	54.0 (6.7)	50.0 (9.1)
Calm VAS (0–100)	70.0 (17.7)	53.4 (12.2) *^,†^
Irritability (0–3)	0.98 (0.29)	1.56 (0.52) *^,†^
Overall risk taking VAS (0–100)	53.1 (4.4)	53.3 (4.2)
Risk and thrill seeking VAS (0–100)	50.6 (6.9)	54.1 (6.0) *^,†^
Self-confidence EVAR (0–100)	54.2 (6.2)	51.0 (8.6)
Need for control EVAR (0–100)	54.5 (9.6)	54.9 (10.1)
Alcohol Hangover Severity Scale (0–10)	0.6 (0.4)	3.8 (1.0) *^,†^
One-item hangover severity (0–100)	0.3 (1.1)	60.3 (20.0) *^,†^

Mean and standard deviation (SD) are shown. Abbreviations: VAS, visual analogue scale; EVAR, Evaluation of Risks Scale. Significant differences (*p* < 0.05) are indicated by * (parametric) and ^†^ (nonparametric).

**Table 2:** Performance outcomes.

Test	Measure	Control	Alcohol
Arrow Flankers	RT (ms)	625.8 (127.8)	693.9 (143.5) *^,†^
	Errors (*n*)	3.4 (6.6)	13.9 (16.5) *^,†^
Serial Sevens	RT (ms)	1948.9 (1938.2)	2055.6 (1339.3)
	Errors (*n*)	3.3 (3.7)	4.4 (4.6)
Continuous Performance	RT (ms)	468.5 (67.5)	506.0 (69.0) *^,†^
	Errors (*n*)	2.4 (3.0)	2.6 (2.5)
Choice Reaction Time	RT (ms)	597.9 (263.0)	705.0 (245.2) *^,†^
	Errors (*n*)	2.8 (2.1)	4.5 (10.6)
Stop Signal Task	RT (ms)	797.3 (115.1)	828.3 (120.6)
	Errors (*n*)	4.2 (1.5)	3.7 (1.3)

Mean and standard deviation (SD) are shown. Abbreviations: RT, reaction time; ms, milliseconds; *n*, number. Significant differences (*p* < 0.05) are indicated by * (parametric) and ^†^ (nonparametric).
